# Risk Scores of Bleeding Complications in Patients on Dual Antiplatelet Therapy: How to Optimize Identification of Patients at Risk of Bleeding after Percutaneous Coronary Intervention

**DOI:** 10.3390/jcm11133574

**Published:** 2022-06-21

**Authors:** Francesco Pelliccia, Felice Gragnano, Vincenzo Pasceri, Arturo Cesaro, Marco Zimarino, Paolo Calabrò

**Affiliations:** 1 Department of Cardiovascular Sciences, Sapienza University, Viale del Policlinico 155, 00166 Rome, Italy; 2 Division of Clinical Cardiology, Azienda Ospedaliera di Rilievo Nazionale ‘Sant’Anna e San Sebastiano’, 81100 Caserta, Italy; gragnano.f@gmail.com (F.G.); arturocesaro@hotmail.it (A.C.); paolo.calabro@unicampania.it (P.C.); 3 Department of Translational Medical Sciences, University of Campania ‘Luigi Vanvitelli’, 80131 Naples, Italy; 4 Interventional Cardiology, San Filippo Neri Hospital, 00135 Rome, Italy; vpasceri@hotmail.com; 5 Institute of Cardiology, “G. d’Annunzio” University, 66100 Chieti, Italy; marco.zimarino@unich.it; 6 Cath Lab, Ospedale Policlinico SS. Annunziata Annunziata Hospital, 66100 Chieti, Italy

**Keywords:** bleeding, dual antiplatelet therapy, percutaneous coronary intervention risk score

## Abstract

Dual antiplatelet therapy (DAPT) with aspirin and a P2Y_12_ receptor inhibitor in patients undergoing percutaneous coronary intervention (PCI) reduces the risk of ischemic events but reduces the risk of ischemic events but increases the risk of bleeding, which in turn is associated with increased morbidity and mortality. With the aim to offer personalized treatment regimens to patients undergoing PCI, much effort has been devoted in the last decade to improve the identification of patients at increased risk of bleeding complications. Several clinical scores have been developed and validated in large populations of patients with coronary artery disease (CAD) and are currently recommended by guidelines to evaluate bleeding risk and individualize the type and duration of antithrombotic therapy after PCI. In clinical practice, these risk scores are conventionally computed at the time of PCI using baseline features and risk factors. Yet, bleeding risk is dynamic and can change over time after PCI, since patients can worsen or improve their clinical status and accumulate comorbidities. Indeed, evidence now exists that the estimated risk of bleeding after PCI can change over time. This concept is relevant, as the inappropriate estimation of bleeding risk, either at the time of revascularization or subsequent follow-up visits, might lead to erroneous therapeutic management. Serial evaluation and recalculation of bleeding risk scores during follow-up can be important in clinical practice to improve the identification of patients at higher risk of bleeding while on DAPT after PCI.

## 1. Introduction

Percutaneous coronary intervention (PCI) is now a class I treatment option in patients with acute coronary syndrome (ACS) and is commonly performed in patients with chronic coronary syndromes (CCS) [1,2]. Since the introduction of PCI in the therapeutic armamentarium, adjunctive antithrombotic therapy with thienopyridine has been shown to play a crucial role in preventing thrombotic complications after PCI [3]. In the last decade, antiplatelet treatment regimens recommended in patients undergoing PCI have changed deeply [4]. Along with the development of new pharmacologic agents, a major determinant of serial changes in guidelines has been the emerging knowledge that bleeding, the most common complication related to antiplatelet treatments, has a major prognostic role [4]. As a matter of fact, dual antiplatelet therapy (DAPT) with aspirin and a P2Y_12_ receptor inhibitor in patients undergoing PCI reduces the risk of ischemic events but increases bleeding complications [5]. With the aim to offer personalized treatment regimens to patients undergoing PCI, much effort in the last decade has been devoted to the identification of patients who are at high risk of bleeding [5]. With this purpose, several clinical risk scores have been developed and validated in large populations of patients with CAD undergoing stent implantation. However, in real-world practice, there is still an urgent need to improve bleeding risk stratification and prediction in patients receiving DAPT [6].

The aim of this review is to sum up current evidence on DAPT in patients with ACS and CCS, provide a comprehensive overview of available bleeding risk scores and algorithms, and highlight recent findings that have the potential to improve the identification of patients at higher risk of bleeding while on DAPT after PCI.

## 2. Current Guidelines on Dual Antiplatelet Therapy after PCI

The standard of care for treating patients undergoing PCI is DAPT [1,2]. After the loading dose, 75–100 mg aspirin should be given to all patients in combination with a P2Y_12_ inhibitor. The choice of a P2Y_12_ inhibitor depends on the clinical presentation (i.e., CCS or ACS) and both the thrombotic and bleeding risks of any given patient. Importantly, evidence now exists that short- and long-term adverse events in patients treated with DAPT are closely associated with both bleeding and ischemic events [6].

**Figure 1:**
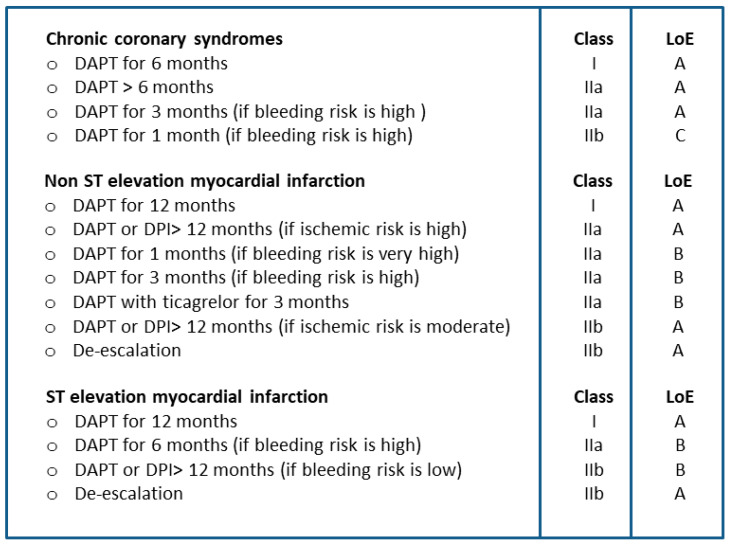
Current recommendations of the European Society of Cardiology about the strategies for antiplatelet treatment after percutaneous coronary intervention. DAPT: dual antiplatelet therapy; DPI: dual pathway inhibition; LoE: level of evidence.

The current recommendations of the European Society of Cardiology (ESC) regarding DAPT are shown in Figure 1 [1,2]. Briefly, DAPT is recommended for six months following elective PCI and 12 months following ACS. In patients with CCS, DAPT can be stopped after 1 or 3 months if a high bleeding risk status is recognized [2]. In patients with ACS, prasugrel and ticagrelor, compared to clopidogrel, significantly reduce the incidence of major adverse cardiovascular events (MACE) [1]. Moreover, the long-term use of low-dose ticagrelor plus aspirin in patients with previous myocardial infarction can improve outcomes compared with aspirin monotherapy, provided that residual ischemic risk is augmented while bleeding risk is low. In patients with and without ST-elevation myocardial infarction, DAPT can be shortened to 6 months (or earlier) if the estimated bleeding risk is high.

Similarly to the ESC recommendations, the latest clinical guideline from the American College of Cardiology and American Heart Association provides an evidence-based approach to the treatment of patients undergoing coronary revascularization [7]. Of note, the American guidelines include the feasibility of a shorter one-to-three-month duration of DAPT after PCI in selected patients to reduce the risk of bleeding compared with the European recommendations of six or twelve months DAPT. Additionally, a short duration of DAPT after PCI in patients with CCS is reasonable to reduce the risk of bleeding events. After consideration of recurrent ischemia and bleeding risks, the selected patients may safely transition to P2Y_12_ inhibitor monotherapy and stop aspirin after 1 to 3 months of DAPT.

## 3. Balancing the Risk of Ischemia and Bleeding after PCI

**Figure 2:**
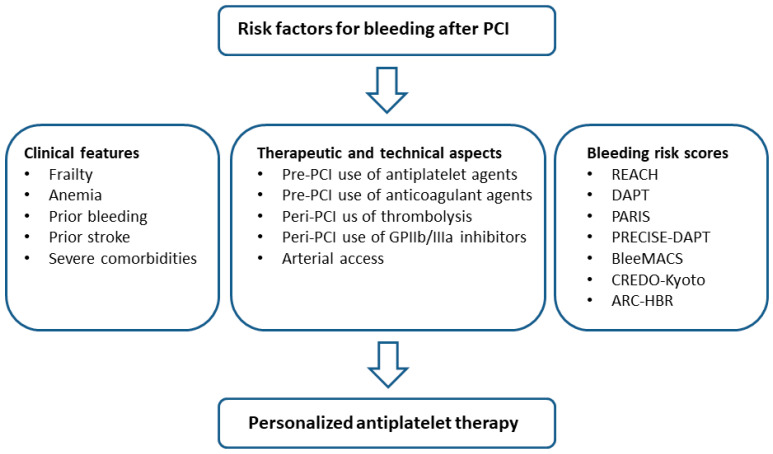
Risk factors to be considered to assess the bleeding risk after percutaneous coronary intervention. ARC-HBR: The Academic Research Consortium for High Bleeding Risk; BleeMACS, bleeding complications in a multicenter registry of patients discharged with diagnosis of acute coronary syndrome; CREDO-Kyoto: coronary revascularization demonstrating outcome study in Kyoto; DAPT: dual antiplatelet therapy; GUSTO, global utilization of streptokinase and TPA for occluded coronary arteries; PARIS, patterns of non-adherence to anti-platelet regimens in stented patients; PCI: percutaneous coronary intervention; PRECISE-DAPT, predicting bleeding complications in patients undergoing stent implantation and subsequent dual anti-platelet therapy; REACH, reduction in atherothrombosis for continued health registry.

Stratification of the individual risks of ischemia and bleeding is of paramount importance for a personalized approach to DAPT after PCI [8]. To this end, a careful evaluation of clinical and angiographic features plays a crucial role [9]. The cornerstone for risk assessment lies in the assessment of the clinical status, which includes patient history and physical examination, as well as the evaluation of comorbidities and laboratory findings. In patients undergoing PCI, angiographic and procedural features play a key role in determining the risk of ischemic recurrences and should be taken into careful consideration [10]. Accordingly, there is general agreement that the optimal strategy for balancing the risk of ischemia and bleeding after PCI lies in an integrated assessment of three key factors: bleeding risk, ischemic risk and responsiveness to an antiplatelet agent (Figure 2) [10]. In this respect, risk algorithms and scores are crucial for weighing the value of individual clinical, laboratory and procedural features. Numerous risk factors that have been proven to be associated with increased bleeding and/or ischemic risks have been used to develop numerical scores and algorithms to offer a prognostic stratification and predict bleeding and/or ischemic events, with the ultimate goal of guiding the choice of type and duration of antiplatelet therapy after PCI [10]. In this regard, recent ESC guidelines recommend the use of thrombotic risk criteria to stratify the risk of future ischemic events and select patients at high or moderate thrombotic risk who might benefit from an intensified antithrombotic treatment. Guidelines also recommend that bleeding risk be a key determinant in defining DAPT duration [1,2]. Of note, several patients are at risk of either bleeding or ischemic complications; when this is the case, the decision making on DAPT duration should be based on the bleeding rather than the ischemic risk [10,11,12,13].

## 4. Risk Stratification of Bleeding Using Scoring Systems

Identification of patients with a high bleeding risk that might benefit from a shorter duration of DAPT can now be achieved by using standardized risk scores or algorithms. Bleeding predictors primarily consider the patient’s characteristics, the complexity of the procedure and the potency of the antithrombotic regimen [10].

For years, no standardized tool has been available to predict long-term bleeding risk for post-PCI patients on DAPT. Among the existing models that have been tested and validated, all were originally developed for alternative purposes and applied to DAPT patients empirically [14,15,16,17,18]. On the one hand, many risk prediction and validation studies have been conducted among patients on warfarin; on the other hand, the risk scores have focused on peri-procedural bleeding, thus failing to inform long-term prescription practices after discharge. Previously proposed risk scores include the HAS-BLED (hypertension, abnormal renal/liver function, stroke, bleeding history or predisposition, labile international normalized ratio, elderly (>65 years), drugs/alcohol concomitantly), which was originally derived for risk prediction of bleeding in anticoagulated patients with vitamin K antagonists [14]; the CHA2DS2-VASc (congestive heart failure, hypertension, age ≥ 75 years, diabetes mellitus, stroke or transient ischemic attack, vascular disease, age 65 to 74 years, sex category), developed for predicting stroke risk in atrial fibrillation [15]; the CRUSADE (can rapid risk stratification of unstable angina patients suppress adverse outcomes with early implementation of the American College of Cardiology/American Heart Association Guidelines), proposed for estimating in-hospital major bleeding; and the ACUITY (acute catheterization and urgent intervention triage strategy), which was developed for mortality risk prediction after myocardial infarction [16,17]. Comparison of these scores in predicting long-term bleeding after PCI revealed a suboptimal discrimination performance, with c-index values ranging between 0.63 and 0.70, thus reflecting the fact that these scores were developed in different settings for different purposes [18].

In the last decade, dedicated risk scores have been developed and validated to address the proper identification of bleeding risk in post-PCI patients (Table 1) [19,20,21,22,23,24,25]. Interestingly, there were specific risk factors in each bleeding risk score, and no single factor was included in all risk scores (Table 2) [19,20,21,22,23,24,25]. Validation studies have shown that these scores are able to identify those patients with a higher risk of long-term bleeding events [10]. The PRECISE-DAPT score (available at http://www.precisedaptscore.com (accessed on 17 June 2022)) was incorporated into the 2017 ESC focused update on DAPT in CAD patients [26] and has spread in clinical practice thereafter. It consists of four continuous parameters (age, creatinine clearance, hemoglobin and white-blood-cell count) and the categorical variable of previous spontaneous bleeding [22]. Recently, the Academic Research Consortium for High Bleeding Risk (ARC-HBR) agreed to propose a novel approach to identify high bleeding risk patients after PCI based on 20 risk criteria (14 major and 6 minor criteria) [25]. This new definition has been retrospectively validated either in the overall population of PCI patients [27,28] or in specific subgroups of interest [29,30]. However, no studies have prospectively validated the role of ARC-HBR criteria to identify subgroups of patients who might benefit most from specific DAPT regimens.

## 5. Reassessment of Bleeding Scores: A Novel Strategy to Improve Risk Factor Characterization

Despite several bleeding prediction scores being currently available and recommended by international guidelines to characterize patients undergoing PCI, the identification of patients at high bleeding risk remains challenging in clinical practice [10]. Indeed, validation studies have shown that the available scores have modest-to-good discrimination ability for bleeding prediction. A possible explanation of the suboptimal performance lies in the fact that CAD is a disease in older adults and is often associated with multiple cardiovascular risk factors and several comorbidities, which can complicate risk prediction in these patients [31]. Of note, comorbidities might develop sometime after PCI, which in turn might increase the risk of bleeding weeks or months after the index procedure [32]. When assessing the risk of bleeding, bleeding scores are conventionally calculated at the time of PCI based on the baseline risk factors, and the outcomes are determined after a follow-up period. Yet, patients’ risk does not remain static but is rather dynamic because patients may accumulate novel comorbidities over time [26]. Therefore, the decision to prolong or abbreviate DAPT duration should be dynamic and reassessed during follow-up.

**Figure 3:**
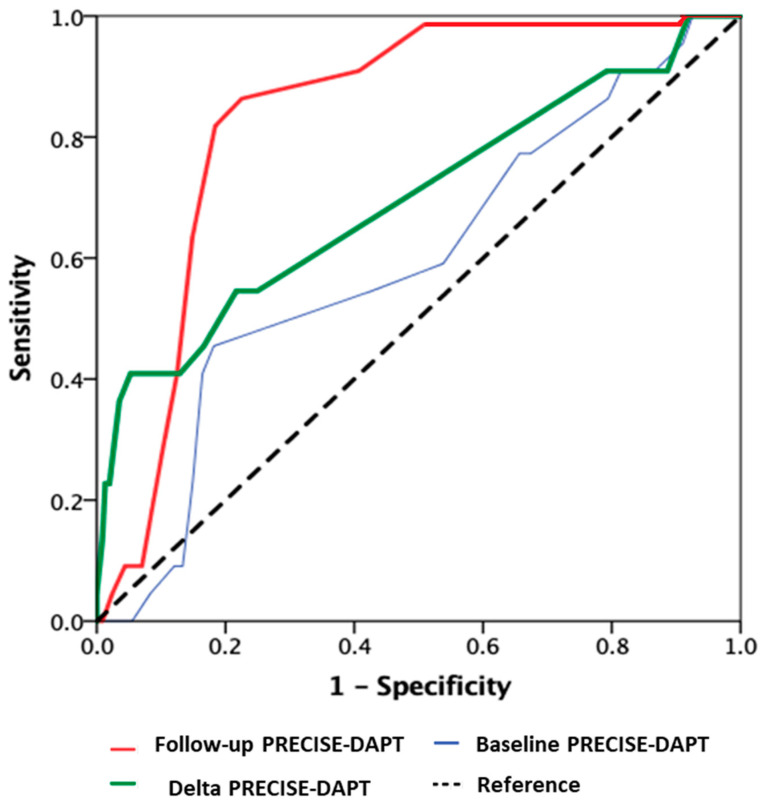
Area under the curve in predicting bleeding in baseline, follow-up and delta PRECISE-DAPT scores, as assessed in patients receiving DAPT included in the RESCORE multicenter prospective registry (reprinted with permission from Ref. [33]. Copyright 2020 Oxford University Press).

These observations are in keeping with the results of the RE-SCORE study, a multicenter registry that sought to investigate the possible prognostic implications of changes in the PRECISE-DAPT score at long-term follow-up [33,34]. The RE-SCORE investigators prospectively evaluated, for the first time, the predictive ability of longitudinal variations in the PRECISE-DAPT score assessed during follow-up in PCI patients treated with DAPT [33]. The main findings of this study were as follows. First, the PRECISE-DAPT score did not remain unchanged with time in a significant subgroup of patients on DAPT. Second, the PRECISE-DAPT score increased more commonly over follow-up in females, as well as in those with multivessel CAD and/or comorbidities. Third, the follow-up PRECISE-DAPT predicted bleeding risk better than the baseline score, particularly in women as compared with men [34]. Of note, the area under the curve of the PRECISE-DAPT score recorded at follow-up was excellent (c-index = 0.84) for the prediction of bleeding events during the subsequent 1-year follow-up, whereas the performance of baseline PRECISE-DAPT appeared modest (c-index = 0.59) (Figure 3).

Overall, the results of the RE-SCORE registry showed that the external validation of the PRECISE-DAPT score calculated at baseline was barely satisfactory, whereas the performance of the score improved when it was reassessed regularly. These findings support the concept that the risk of bleeding is not static and that the PRECISE-DAPT score can do well in the real world only if the score is recalculated over follow-up. Apart from age and previous spontaneous bleeding, three determinants of the score can fluctuate with time. Creatinine clearance can decrease after PCI, as renal function might progressively deteriorate as a consequence of acute kidney injury [35], post-procedural low-grade inflammation [36], activation of the renin angiotensin aldosterone system [37] or adverse effects of pharmacologic agents (i.e., nonsteroidal anti-inflammatory drugs) [38]. Anemia is a frequent comorbidity in patients with CAD undergoing PCI and is associated with increased morbidity and mortality [11]. Post-procedural anemia might result from the presence of chronic unrecognized bleeding, which might worsen after the initiation of DAPT and, in turn, could increase the future risk of bleeding and mortality [39]. Unfortunately, the presence of a small decline in hemoglobin is often regarded as ‘not severe’ and dismissed [40], and subtle changes in hemoglobin while on DAPT and their correlation with the prognosis have never been previously taken into consideration in the prediction of bleeding in the long term. Finally, white blood cell is an easily obtained surrogate marker of inflammation [41]. The significance of an elevated white blood cell after PCI remains despite the improvements in medical therapy [41]. In fact, the possibility exists that chronic inflammatory responses to stent polymers play a role in the underlying pathophysiology of increased white-blood-cell count [42] and its precursors [43], which have been associated with a poorer long-term outcome [44].

The findings from the RE-SCORE investigators underscore the concept that patients who are given DAPT after PCI are potentially at high risk and therefore require attention and close surveillance over time by clinical and laboratory reassessment [45]. To this end, further studies using other risk score systems are needed to confirm whether the recalculation of bleeding risk yields a better performance.

## 6. Conclusions

Several risk scores are currently recommended by the guidelines to evaluate bleeding risk and individualize the type and duration of antithrombotic therapy after PCI. In clinical practice, these risk scores are generally assessed at the time of PCI using baseline features and risk factors. Yet, bleeding risk is dynamic and can significantly change over time. This concept is relevant, as the inappropriate estimation of bleeding risk, either at the time of revascularization or subsequent follow-up visits, could lead to erroneous therapeutic management. With this respect, recent findings shed light on a novel predictive strategy that might enhance the proper identification of patients at higher risk of bleeding. The evidence that bleeding scores are dynamic underscores that the estimated risk of bleeding after PCI can change with time and should therefore be regularly reassessed. Frequent evaluation and recalculation of bleeding scores should be implemented in clinical practice to potentially improve the identification of high bleeding patients after PCI.

## Figures and Tables

**Table 1:** Scores specifically derived for assessing long-term bleeding risk in patients taking dual antiplatelet therapy after percutaneous coronary intervention.

	REACH	DAPT	PARIS	PRECISE-DAPT	BleeMACS	Credo-Kyoto	ARC-HBR
Year	2010	2016	2016	2017	2018	2018	2019
**Derivation cohort**	REACH Registry	DAPT RCT	PARIS Registry	Eight RCTs pooled	BleeMACS Registry	CREDO-Kyoto Registry	The Academic Research Consortium for High Bleeding Risk
**No. of patients**	56,616	11,148	4190	14,963	15,401	4778	NA
**Validation cohort**	CHARISMA	PROTECT	ADAPT-DES	PLATO and Bern PCI Registry	SWEDEHEART	RESET and NEXT	Bern PCI Registry
**No. of patients**	15,603	8136	8130	8595 and 6172	96,239	12,223	16,580
**Country**	Europe, USA		Europe, USA	Europe, Korea, Brazil, Israel		Japan	Europe, USA
**Bleeding outcome**	2-year serious bleeding	Major bleeding 12 to 30 months after PCI	2-year serious bleeding	Out-of-hospital bleeding at median F/U of 552 days	1-year serious spontaneous bleeding	2-year major bleeding	1-year major bleeding
**Bleeding criteria**	Protocol defined	GUSTO moderate or severe	BARC 3 or 5	TIMI major or minor bleeding	Protocol defined	GUSTO moderate or severe	BARC 3 or 5
**Score range**	0 to 23	−2 to 10	0 to 14	0 to 100	0 to 80	0 to 11	NA

ADAPT-DES, assessment of dual antiplatelet therapy with drug-eluting stents; ARC-HBR: The Academic Research Consortium for High Bleeding Risk; BARC, Bleeding Academic Research Consortium; BleeMACS, Bleeding complications in a multicenter registry of patients discharged with diagnosis of acute coronary syndrome; CHARISMA, clopidogrel for high atherothrombotic risk and ischemic stabilization, management and avoidance; CREDO-Kyoto: coronary revascularization demonstrating outcome study in Kyoto; DAPT: dual antiplatelet therapy; GUSTO, global utilization of streptokinase and TPA for occluded coronary arteries; HBR, high bleeding risk; NEXT: Nobori Biolimus-eluting versus Xience/Promus Everolimus-eluting stent trial; PARIS, patterns of non-adherence to anti-platelet regimens in stented patients; PCI, percutaneous coronary intervention; PLATO, platelet inhibition and patient outcomes; PRECISEDAPT, predicting bleeding complications in patients undergoing stent implantation and subsequent dual anti-platelet therapy; PROTECT, patient-related outcomes with endeavor versus cypher stenting trial; REACH, reduction in atherothrombosis for continued health registry; RCT: randomized controlled trial; RESET: randomized evaluation of Sirolimus-eluting versus Everolimus-eluting stent trial; SWEDEHEART: Swedish web system for enhancement and development of evidence-based care in heart disease evaluated according to recommended therapies; TIMI, thrombolysis in myocardial infarction.

**Table 2:** Variables included in scores developed and validated to assess long-term bleeding risk in patients taking dual antiplatelet therapy after percutaneous coronary intervention.

	REACH	DAPT	PARIS	PRECISE-DAPT	BleeMACS	Credo-Kyoto	ARC-HBR
**Age**	x	x	x	x	x	x	x
**Body Mass Index**			x				
**Oral anticoagulants**	x		x				x
**Antiplatelet therapy**	x	x					
**Anemia**			x	x	x	x	x
**White cell count**				x			x
**Thrombocytopenia**							x
**Atrial fibrillation**						x	
**Kidney disease**		x	x	x	x	x	x
**Liver cirrhosis**							x
**Peripheral artery disease**	x	x			x	x	
**Chronic heart failure**	x					x	
**Prior bleeding**	x			x	x		x
**Prior major stroke**							x
**Prior trauma or surgery**							x
**Prior or active malignancy**					x		
**Chronic total occlusion**						x	
**Diabetes**	x					x	
**Smoking**	x		x				
**Hypertension**	x	x			x		
**Hypercholesterolemia**	x						

ARC-HBR: The Academic Research Consortium for High Bleeding Risk; BleeMACS, bleeding complications in a multicenter registry of patients discharged with diagnosis of acute coronary syndrome; CREDO-Kyoto: coronary revascularization demonstrating outcome study in Kyoto; DAPT: dual antiplatelet therapy; GUSTO, global utilization of streptokinase and TPA for occluded coronary arteries; PARIS, patterns of non-adherence to anti-platelet regimens in stented patients; PRECISE-DAPT, Predicting bleeding complications in patients undergoing stent implantation and subsequent dual anti-platelet therapy; REACH, reduction in atherothrombosis for continued health registry.

## Data Availability

Data sharing not applicable. No new data were created or analyzed in this study. Data sharing is not applicable to this review article.
